# Draft Genome Sequence of *Streptococcus parauberis* Strain SK-417, Isolated from Diseased *Sebastes ventricosus* in Kagoshima, Japan

**DOI:** 10.1128/genomeA.00453-14

**Published:** 2014-05-22

**Authors:** Kazuki Oguro, Jin Yamane, Takeshi Yamamoto, Kouhei Ohnishi, Syun-ichirou Oshima, Masayuki Imajoh

**Affiliations:** a Laboratory of Fish Disease, Faculty of Agriculture, Kochi University, Nankoku, Kochi, Japan; b Azuma-cho Fisheries Cooperative Association, Nagashima, Izumi, Kagoshima, Japan; c Research Institute of Molecular Genetics, Kochi University, Nankoku, Kochi, Japan; d Graduate School of Kuroshio Science, Kochi University, Nankoku, Kochi, Japan

## Abstract

*Streptococcus parauberis* strain SK-417 was isolated from the brain of a diseased *Sebastes ventricosus*, collected from an aquaculture farm in April 2013 in Kagoshima Prefecture, Japan. The draft genome sequence, obtained with a 454 GS Junior sequencing system, consists of 33 large contigs of >500 bp, totaling 1,958,836 bp, and has a G+C content of 35.4%.

## GENOME ANNOUNCEMENT

*Streptococcus parauberis* is an alpha-hemolytic Gram-positive coccoid bacterium belonging to the *Streptococcaceae* family. This bacterium is known to cause mastitis in cows (1) and streptococcosis in fish (2–5). Streptococcosis caused by *S. parauberis* is a major disease in cultured olive flounder (*Paralichthys olivaceus*) in western Japan (6). Japanese isolates have been classified into two serotypes, I and II, based on antigenic differences in their capsular polysaccharide (4). Nho et al. (7) determined the whole-genome sequences of *S. parauberis* serotypes I and II strains and compared them with that of a Korean strain (8), which revealed differentiation for phage resistance between the two serotype strains and for carbon source utilization between the Japanese and Korean strains.

A diseased *Sebastes ventricosus* was collected from an aquaculture farm in April 2013 in Kagoshima Prefecture, which is located in the southernmost part of Kyushu Island, Japan. Gram-positive cocci were isolated from the brain of the fish. We identified them as *S. parauberis* by PCR assay using published species-specific primers (9). Strain SK-417 was cultured by shaking (160 rpm) in 10 ml of brain heart infusion supplemented with 2% (wt/vol) NaCl for 24 h at 27° C. The bacterial cells were pelleted by centrifugation at 2,300 × *g* for 15 min, and the genomic DNA was extracted and purified with a Qiagen Genomic-tip 500/G kit and a genomic DNA buffer set (Qiagen), according to the manufacturer’s instructions. Genome sequencing was performed on a 454 GS Junior system (Roche), which generated a total of 130,423 reads. The sequencing reads were assembled with GS *de novo* Assembler version 2.9 software (Roche). The assembly consists of 33 contigs (>500 bp), with an *N*_50_ contig size of 107,516 bp and a G+C content of 35.4%. The total size is 1,958,836 bp. The draft genome sequence of *S. parauberis* strain SK-417 was annotated using the Microbial Genome Annotation Pipeline that utilizes MetaGeneAnnotator (10), RNAmmer (11), tRNAScan-SE (12), and BLAST (13), yielding a total of 1,950 protein-coding sequences (CDS), 35 tRNA genes, and 3 rRNA operons. Furthermore, the Rapid Annotations using Subsystems Technology (RAST) server (14) was used. The annotation identified 1,022 CDS in RAST subsystems, of which 34 corresponded to hypothetical proteins. Among the subsystems, six subsystem features for photosynthesis, phages, prophages, transposable elements and plasmids, iron acquisition and metabolism, secondary metabolism, regulons, and nitrogen metabolism were absent. It was reported previously that 30 CDS in the genome of *S. parauberis* serotype I strain were categorized as phages, prophages, transposable elements, and plasmids (7), indicating genetic differentiation between the two strains, SK-417 and serotype I. On the other hand, the genome of *S. parauberis* strain SK-417 contains a gene encoding a transcriptional antiterminator similar to that found in the genome of *S. parauberis* serotype I strain but not the serotype II and Korean strains (7). Our findings may be important for understanding genetic variation in *S. parauberis* in Japan.

### Nucleotide sequence accession numbers.

The draft genome sequence of *S. parauberis* strain SK-417 has been deposited in GenBank under the accession no. BAWT00000000. The version described in this paper is version BAWT01000000.
